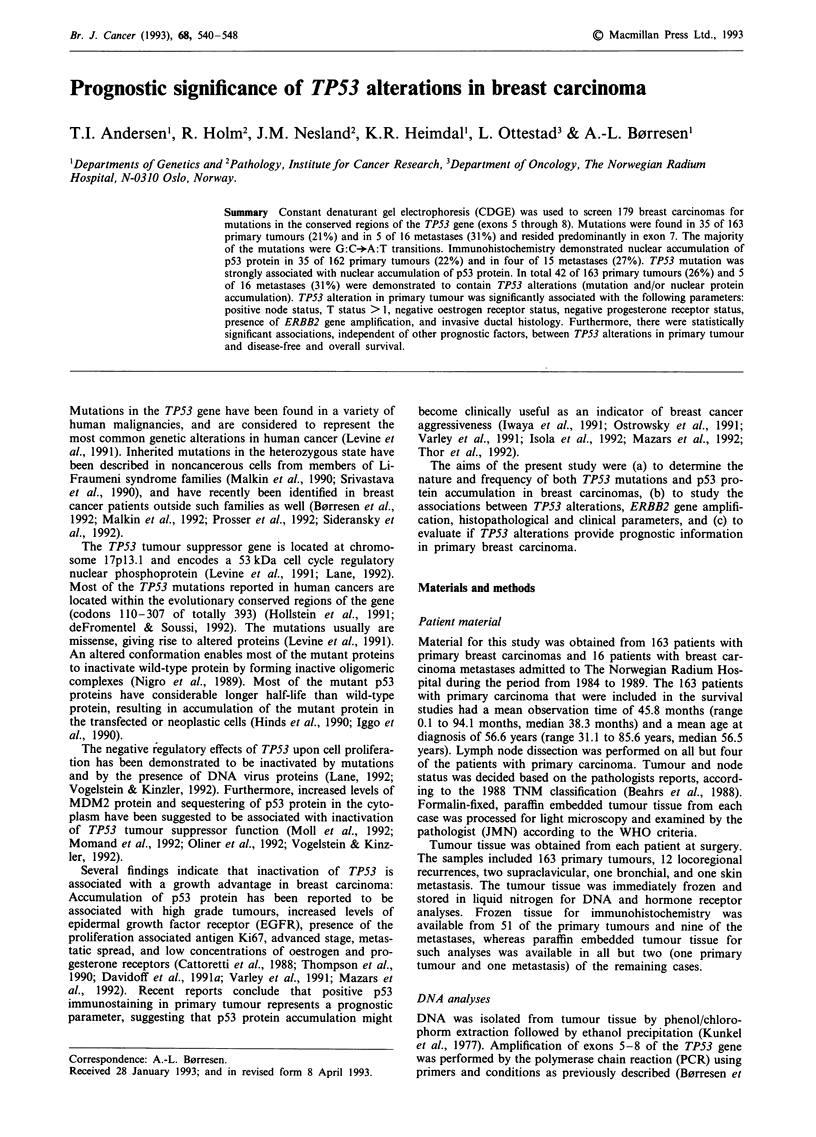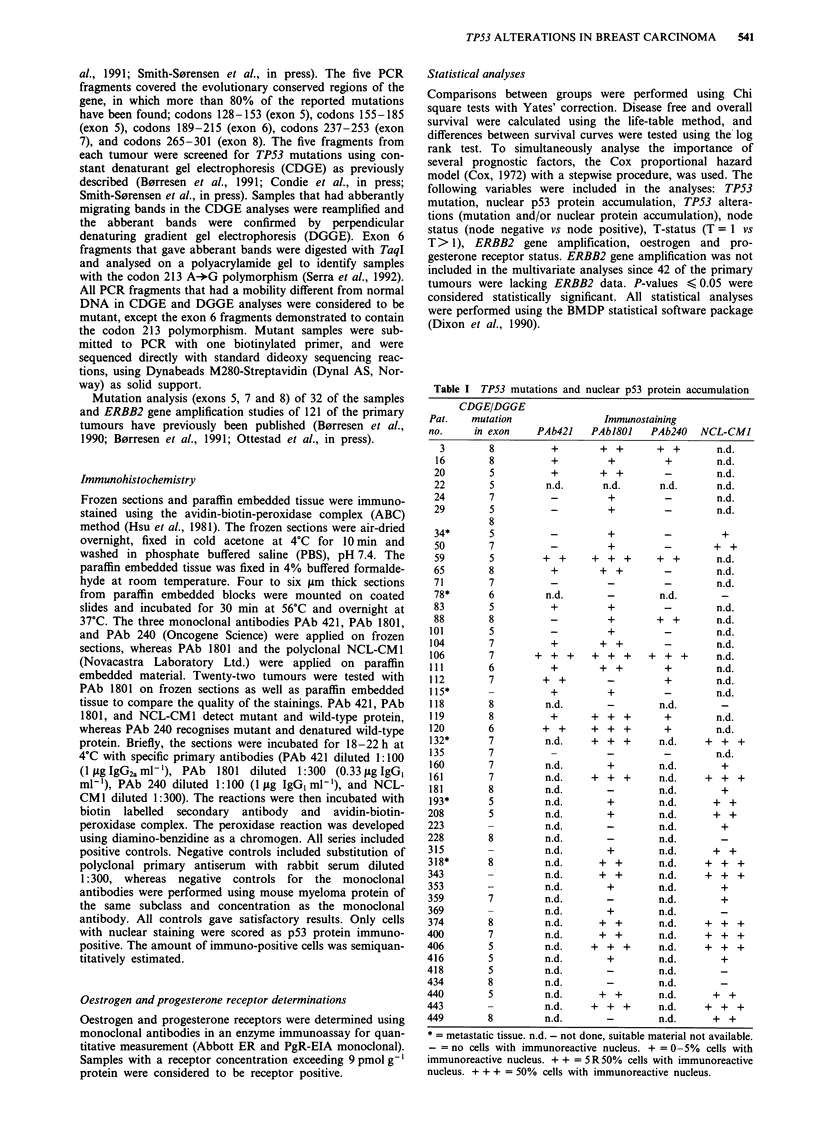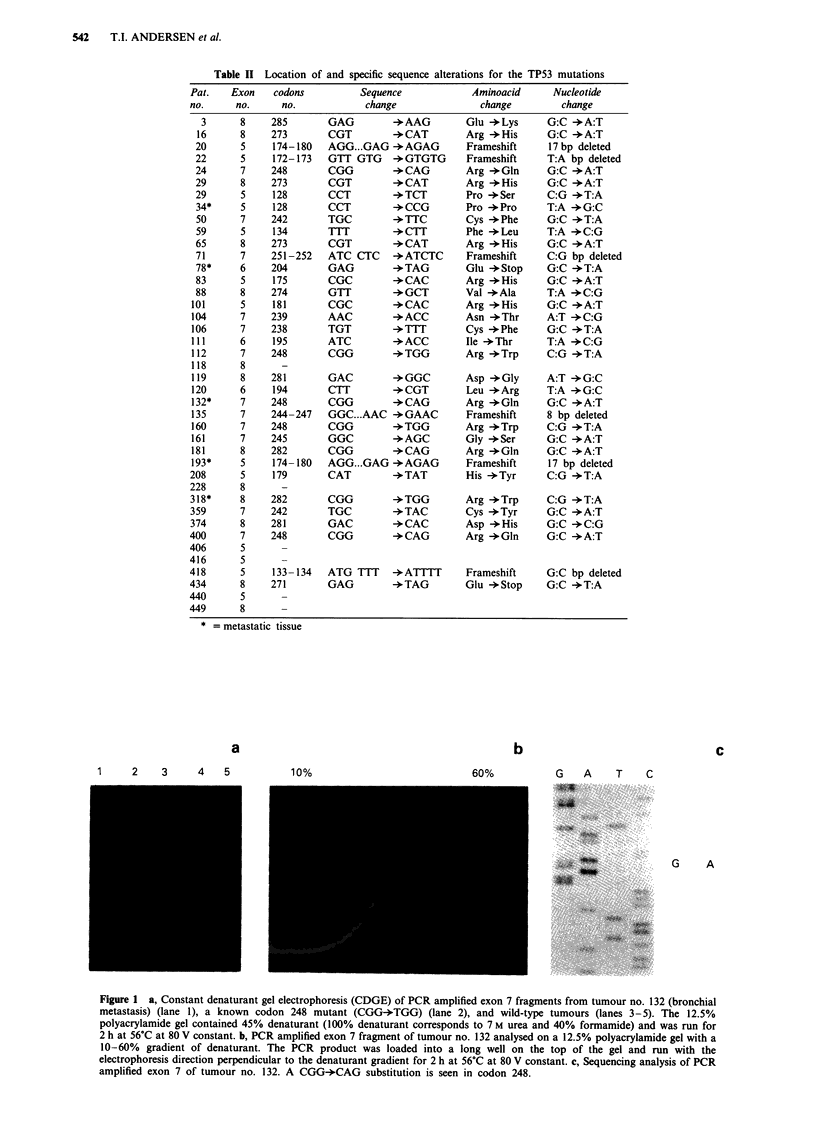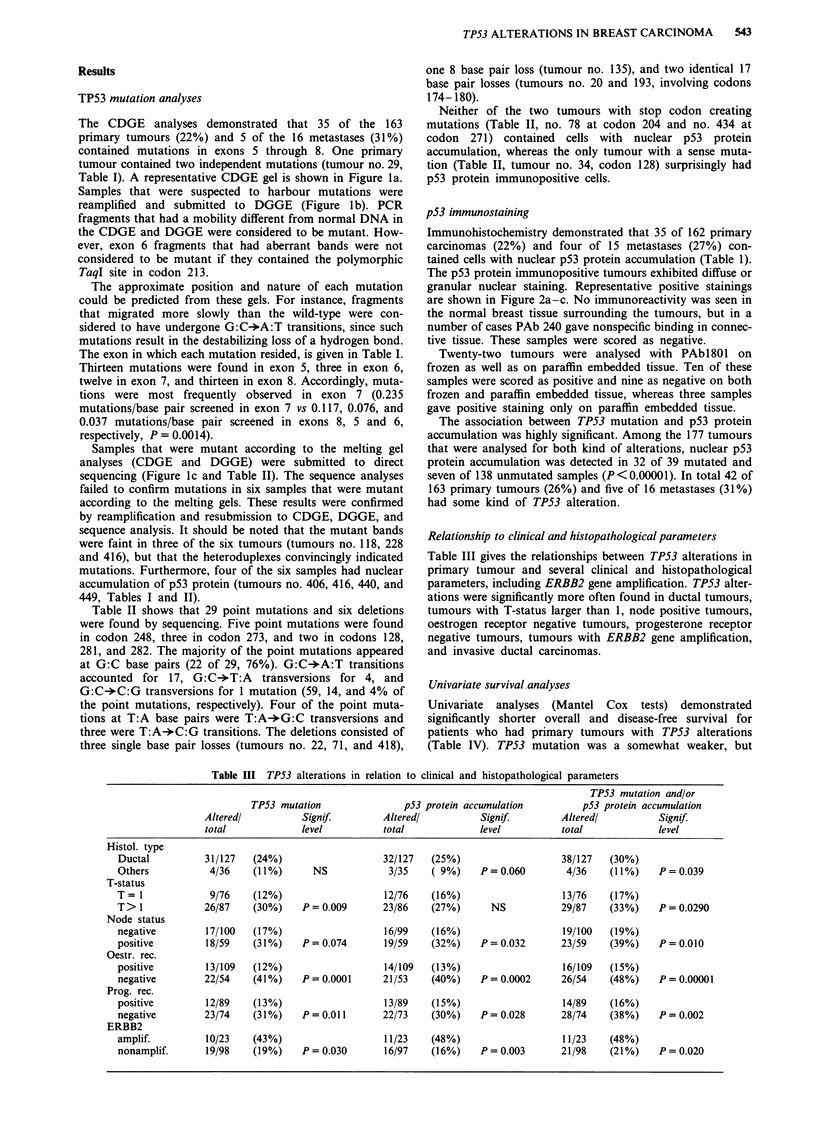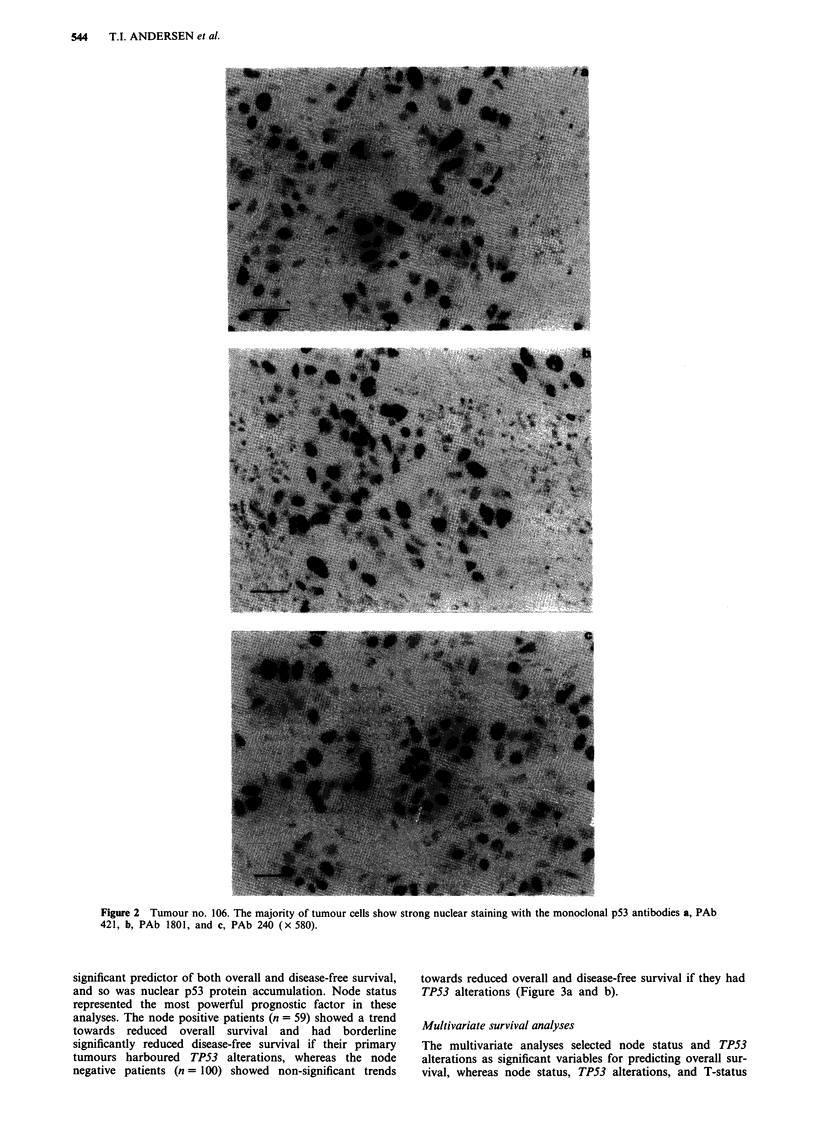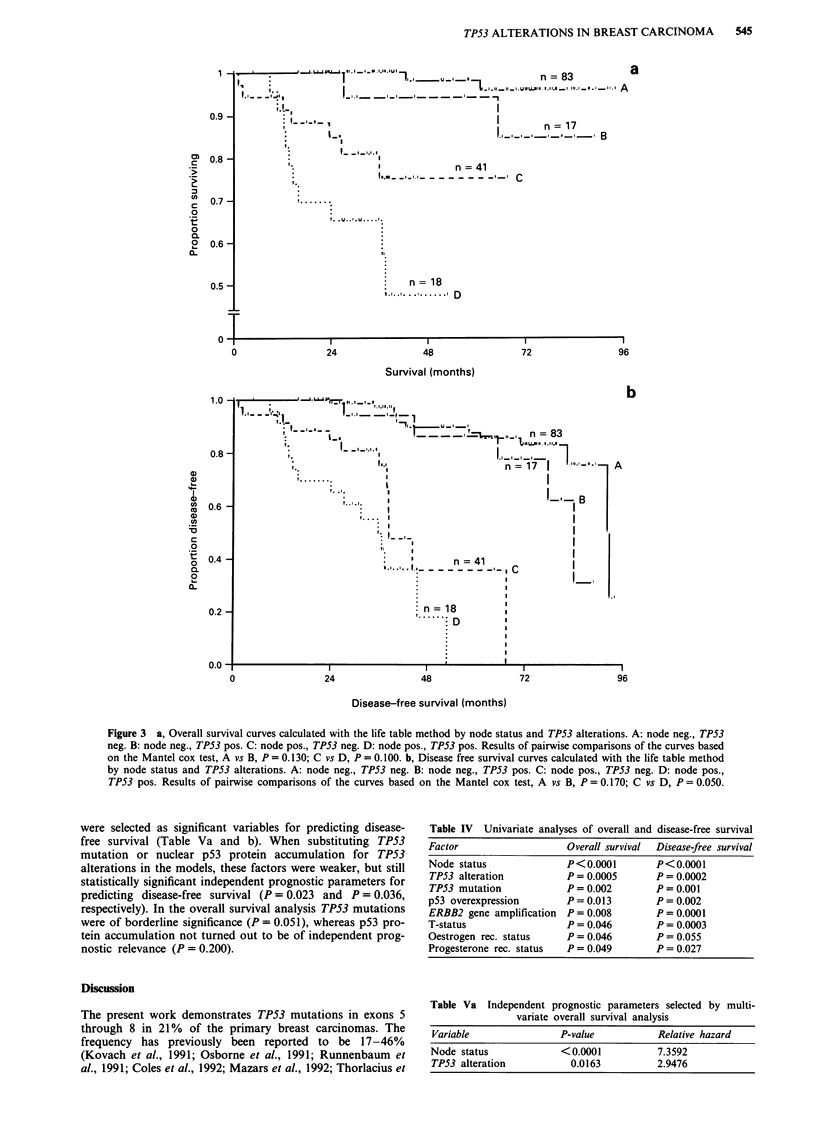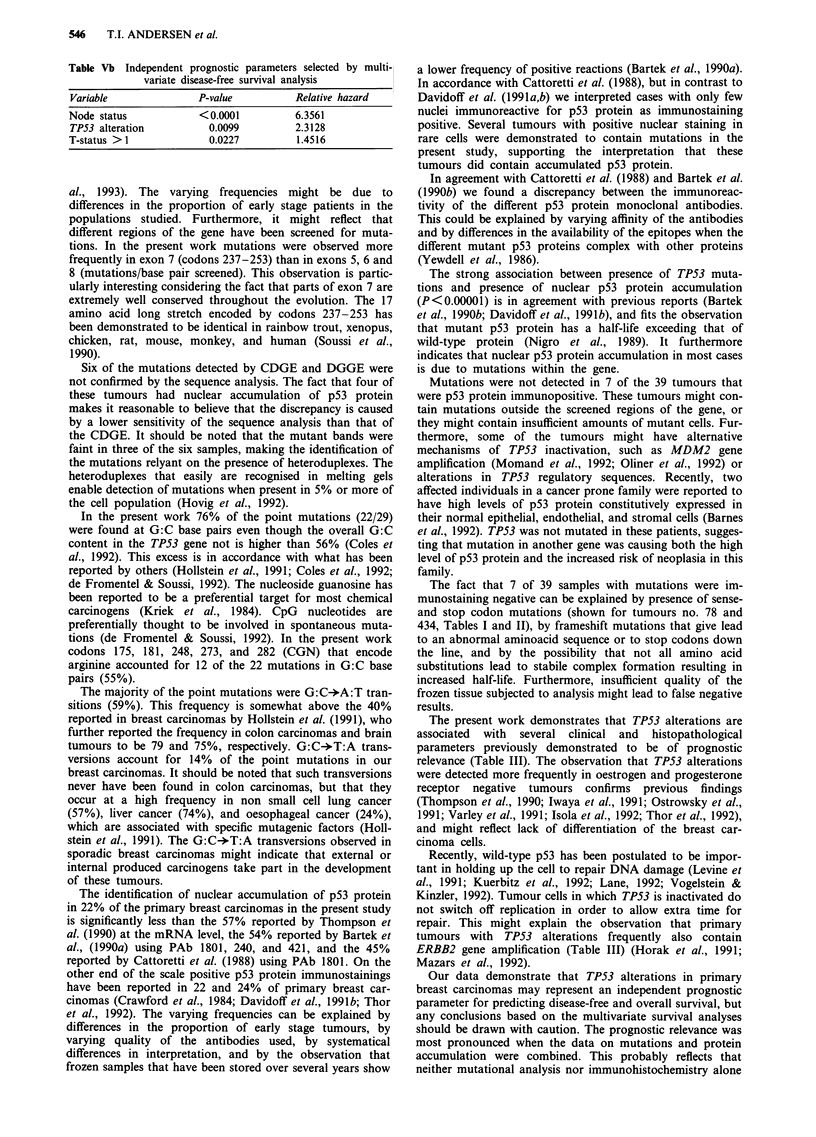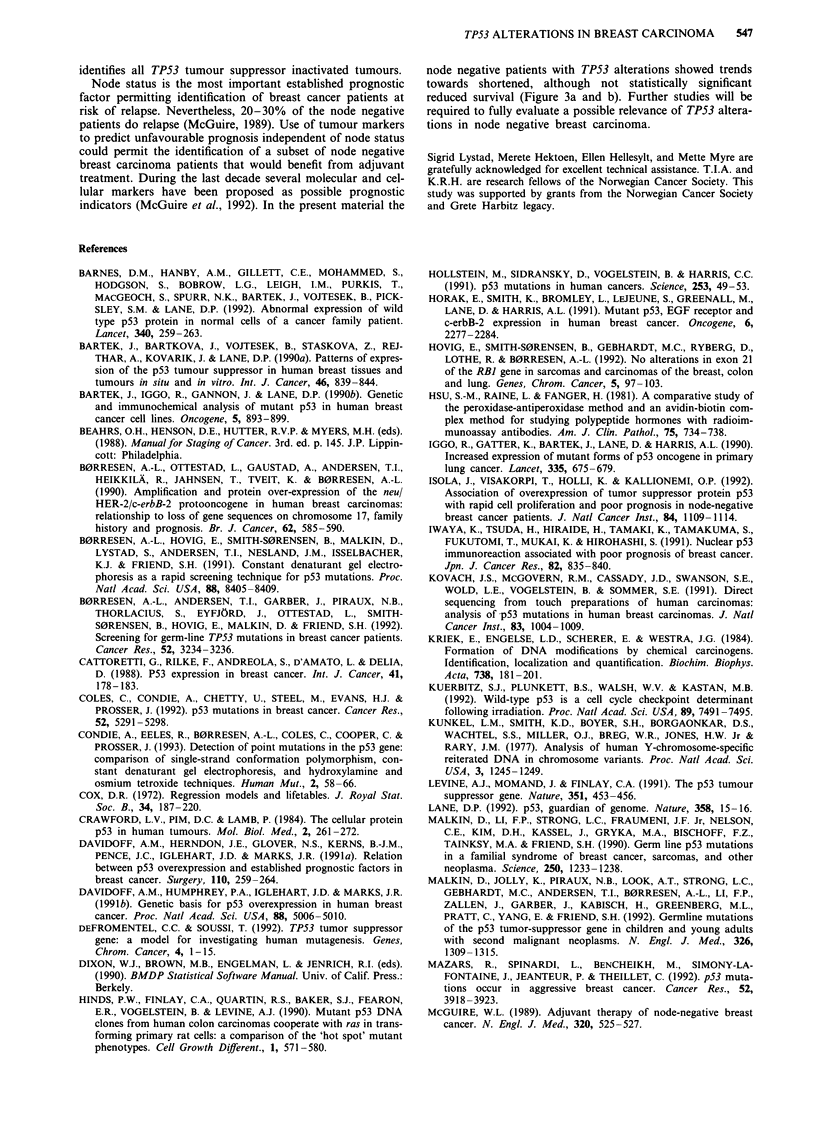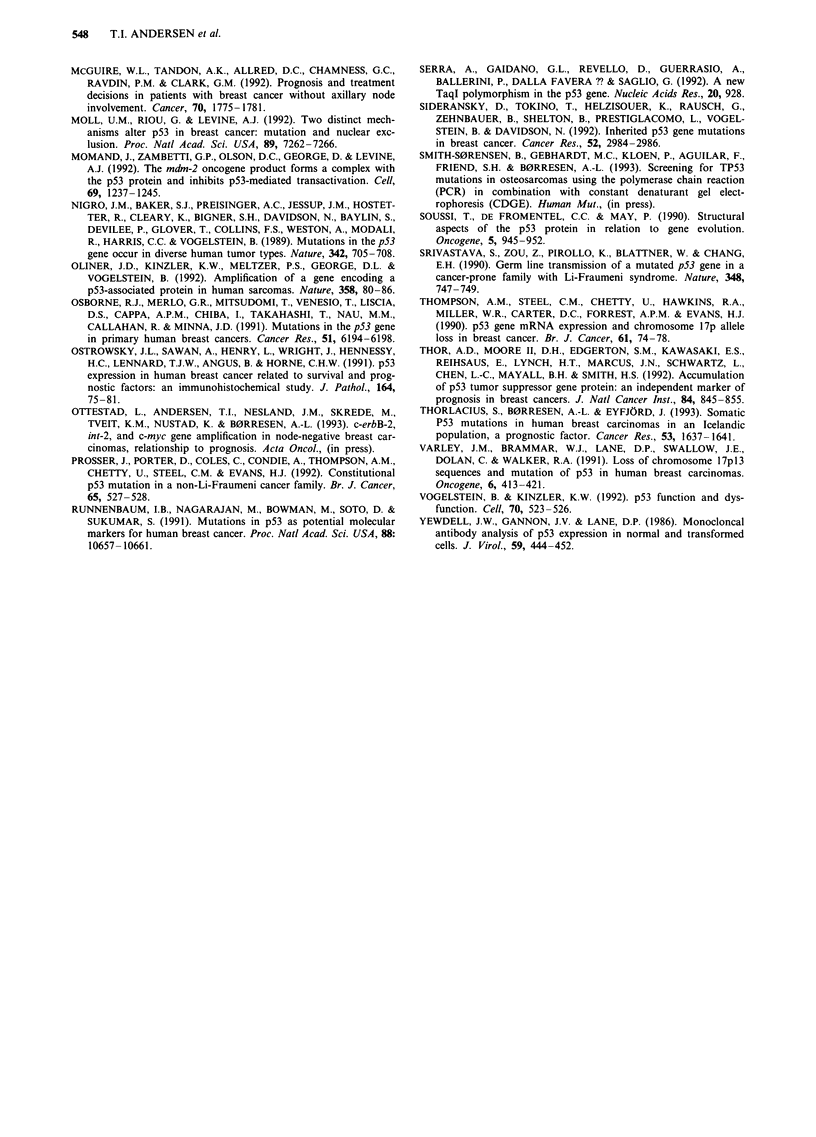# Prognostic significance of TP53 alterations in breast carcinoma.

**DOI:** 10.1038/bjc.1993.383

**Published:** 1993-09

**Authors:** T. I. Andersen, R. Holm, J. M. Nesland, K. R. Heimdal, L. Ottestad, A. L. Børresen

**Affiliations:** Department of Genetics, Norwegian Radium Hospital, Oslo.

## Abstract

**Images:**


Br. J. Cancer (1993), 68, 540-548 © Macmillan Press Ltd., 1993

### Prognostic significance of *TP53* alterations in breast carcinoma

T.I. Andersen[1], R. Holm[2], J.M. Nesland[2], K.R. Heimdal[1], L. Ottestad[3] & A.-L. Børresen[1]

[1]*Departments of Genetics and* [2]*Pathology, Institute for Cancer Research,* [3]*Department of Oncology, The Norwegian Radium Hospital, N-0310 Oslo, Norway.*

**Summary** Constant denaturant gel electrophoresis (CDGE) was used to screen 179 breast carcinomas for mutations in the conserved regions of the *TP53* gene (exons 5 through 8). Mutations were found in 35 of 163 primary tumours (21%) and in 5 of 16 metastases (31%) and resided predominantly in exon 7. The majority of the mutations were G:C→A:T transitions. Immunohistochemistry demonstrated nuclear accumulation of p53 protein in 35 of 162 primary tumours (22%) and in four of 15 metastases (27%). *TP53* mutation was strongly associated with nuclear accumulation of p53 protein. In total 42 of 163 primary tumours (26%) and 5 of 16 metastases (31%) were demonstrated to contain *TP53* alterations (mutation and/or nuclear protein accumulation). *TP53* alteration in primary tumour was significantly associated with the following parameters: positive node status, T status $> 1$, negative oestrogen receptor status, negative progesterone receptor status, presence of *ERBB2* gene amplification, and invasive ductal histology. Furthermore, there were statistically significant associations, independent of other prognostic factors, between *TP53* alterations in primary tumour and disease-free and overall survival.

Mutations in the *TP53* gene have been found in a variety of human malignancies, and are considered to represent the most common genetic alterations in human cancer (Levine *et al.*, 1991). Inherited mutations in the heterozygous state have been described in noncancerous cells from members of Li-Fraumeni syndrome families (Malkin *et al.*, 1990; Srivastava *et al.*, 1990), and have recently been identified in breast cancer patients outside such families as well (Børresen *et al.*, 1992; Malkin *et al.*, 1992; Prosser *et al.*, 1992; Sideransky *et al.*, 1992).

The *TP53* tumour suppressor gene is located at chromosome 17p13.1 and encodes a 53 kDa cell cycle regulatory nuclear phosphoprotein (Levine *et al.*, 1991; Lane, 1992). Most of the *TP53* mutations reported in human cancers are located within the evolutionary conserved regions of the gene (codons 110-307 of totally 393) (Hollstein *et al.*, 1991; deFromentel & Soussi, 1992). The mutations usually are missense, giving rise to altered proteins (Levine *et al.*, 1991). An altered conformation enables most of the mutant proteins to inactivate wild-type protein by forming inactive oligomeric complexes (Nigro *et al.*, 1989). Most of the mutant p53 proteins have considerable longer half-life than wild-type protein, resulting in accumulation of the mutant protein in the transfected or neoplastic cells (Hinds *et al.*, 1990; Iggo *et al.*, 1990).

The negative regulatory effects of *TP53* upon cell proliferation has been demonstrated to be inactivated by mutations and by the presence of DNA virus proteins (Lane, 1992; Vogelstein & Kinzler, 1992). Furthermore, increased levels of MDM2 protein and sequestering of p53 protein in the cytoplasm have been suggested to be associated with inactivation of *TP53* tumour suppressor function (Moll *et al.*, 1992; Momand *et al.*, 1992; Oliner *et al.*, 1992; Vogelstein & Kinzler, 1992).

Several findings indicate that inactivation of *TP53* is associated with a growth advantage in breast carcinoma: Accumulation of p53 protein has been reported to be associated with high grade tumours, increased levels of epidermal growth factor receptor (EGFR), presence of the proliferation associated antigen Ki67, advanced stage, metastatic spread, and low concentrations of oestrogen and progesterone receptors (Cattoretti *et al.*, 1988; Thompson *et al.*, 1990; Davidoff *et al.*, 1991*a*; Varley *et al.*, 1991; Mazars *et al.*, 1992). Recent reports conclude that positive p53 immunostaining in primary tumour represents a prognostic parameter, suggesting that p53 protein accumulation might become clinically useful as an indicator of breast cancer aggressiveness (Iwaya *et al.*, 1991; Ostrowsky *et al.*, 1991; Varley *et al.*, 1991; Isola *et al.*, 1992; Mazars *et al.*, 1992; Thor *et al.*, 1992).

The aims of the present study were (a) to determine the nature and frequency of both *TP53* mutations and p53 protein accumulation in breast carcinomas, (b) to study the associations between *TP53* alterations, *ERBB2* gene amplification, histopathological and clinical parameters, and (c) to evaluate if *TP53* alterations provide prognostic information in primary breast carcinoma.

### Materials and methods

### *Patient material*

Material for this study was obtained from 163 patients with primary breast carcinomas and 16 patients with breast carcinoma metastases admitted to The Norwegian Radium Hospital during the period from 1984 to 1989. The 163 patients with primary carcinoma that were included in the survival studies had a mean observation time of 45.8 months (range 0.1 to 94.1 months, median 38.3 months) and a mean age at diagnosis of 56.6 years (range 31.1 to 85.6 years, median 56.5 years). Lymph node dissection was performed on all but four of the patients with primary carcinoma. Tumour and node status was decided based on the pathologists reports, according to the 1988 TNM classification (Beahrs *et al.*, 1988). Formalin-fixed, paraffin embedded tumour tissue from each case was processed for light microscopy and examined by the pathologist (JMN) according to the WHO criteria.

Tumour tissue was obtained from each patient at surgery. The samples included 163 primary tumours, 12 locoregional recurrences, two supraclavicular, one bronchial, and one skin metastasis. The tumour tissue was immediately frozen and stored in liquid nitrogen for DNA and hormone receptor analyses. Frozen tissue for immunohistochemistry was available from 51 of the primary tumours and nine of the metastases, whereas paraffin embedded tumour tissue for such analyses was available in all but two (one primary tumour and one metastasis) of the remaining cases.

### *DNA analyses*

DNA was isolated from tumour tissue by phenol/chlorophorm extraction followed by ethanol precipitation (Kunkel *et al.*, 1977). Amplification of exons 5-8 of the *TP53* gene was performed by the polymerase chain reaction (PCR) using primers and conditions as previously described (Børresen *et*

Correspondence: A.-L. Børresen.
Received 28 January 1993; and in revised form 8 April 1993.

*al.*, 1991; Smith-Sørensen *et al.*, in press). The five PCR fragments covered the evolutionary conserved regions of the gene, in which more than 80% of the reported mutations have been found; codons 128–153 (exon 5), codons 155–185 (exon 5), codons 189–215 (exon 6), codons 237–253 (exon 7), and codons 265–301 (exon 8). The five fragments from each tumour were screened for *TP53* mutations using constant denaturant gel electrophoresis (CDGE) as previously described (Børresen *et al.*, 1991; Condie *et al.*, in press; Smith-Sørensen *et al.*, in press). Samples that had abberantly migrating bands in the CDGE analyses were reamplified and the abberant bands were confirmed by perpendicular denaturing gradient gel electrophoresis (DGGE). Exon 6 fragments that gave abberant bands were digested with *Taq*I and analysed on a polyacrylamide gel to identify samples with the codon 213 A→G polymorphism (Serra *et al.*, 1992). All PCR fragments that had a mobility different from normal DNA in CDGE and DGGE analyses were considered to be mutant, except the exon 6 fragments demonstrated to contain the codon 213 polymorphism. Mutant samples were submitted to PCR with one biotinylated primer, and were sequenced directly with standard dideoxy sequencing reactions, using Dynabeads M280-Streptavidin (Dynal AS, Norway) as solid support.

Mutation analysis (exons 5, 7 and 8) of 32 of the samples and *ERBB2* gene amplification studies of 121 of the primary tumours have previously been published (Børresen *et al.*, 1990; Børresen *et al.*, 1991; Ottestad *et al.*, in press).

### *Immunohistochemistry*

Frozen sections and paraffin embedded tissue were immunostained using the avidin-biotin-peroxidase complex (ABC) method (Hsu *et al.*, 1981). The frozen sections were air-dried overnight, fixed in cold acetone at 4°C for 10 min and washed in phosphate buffered saline (PBS), pH 7.4. The paraffin embedded tissue was fixed in 4% buffered formaldehyde at room temperature. Four to six μm thick sections from paraffin embedded blocks were mounted on coated slides and incubated for 30 min at 56°C and overnight at 37°C. The three monoclonal antibodies PAb 421, PAb 1801, and PAb 240 (Oncogene Science) were applied on frozen sections, whereas PAb 1801 and the polyclonal NCL-CM1 (Novacastra Laboratory Ltd.) were applied on paraffin embedded material. Twenty-two tumours were tested with PAb 1801 on frozen sections as well as paraffin embedded tissue to compare the quality of the stainings. PAb 421, PAb 1801, and NCL-CM1 detect mutant and wild-type protein, whereas PAb 240 recognises mutant and denatured wild-type protein. Briefly, the sections were incubated for 18–22 h at 4°C with specific primary antibodies (PAb 421 diluted 1:100 (1 $\mu g\ IgG_{2a}\ ml^{-1}$), PAb 1801 diluted 1:300 (0.33 $\mu g\ IgG_1\ ml^{-1}$), PAb 240 diluted 1:100 (1 $\mu g\ IgG_1\ ml^{-1}$), and NCL-CM1 diluted 1:300). The reactions were then incubated with biotin labelled secondary antibody and avidin-biotin-peroxidase complex. The peroxidase reaction was developed using diamino-benzidine as a chromogen. All series included positive controls. Negative controls included substitution of polyclonal primary antiserum with rabbit serum diluted 1:300, whereas negative controls for the monoclonal antibodies were performed using mouse myeloma protein of the same subclass and concentration as the monoclonal antibody. All controls gave satisfactory results. Only cells with nuclear staining were scored as p53 protein immunopositive. The amount of immuno-positive cells was semiquantitatively estimated.

### *Oestrogen and progesterone receptor determinations*

Oestrogen and progesterone receptors were determined using monoclonal antibodies in an enzyme immunoassay for quantitative measurement (Abbott ER and PgR-EIA monoclonal). Samples with a receptor concentration exceeding 9 $pmol\ g^{-1}$ protein were considered to be receptor positive.

### *Statistical analyses*

Comparisons between groups were performed using Chi square tests with Yates' correction. Disease free and overall survival were calculated using the life-table method, and differences between survival curves were tested using the log rank test. To simultaneously analyse the importance of several prognostic factors, the Cox proportional hazard model (Cox, 1972) with a stepwise procedure, was used. The following variables were included in the analyses: *TP53* mutation, nuclear p53 protein accumulation, *TP53* alterations (mutation and/or nuclear protein accumulation), node status (node negative *vs* node positive), T-status (T = 1 *vs* T > 1), *ERBB2* gene amplification, oestrogen and progesterone receptor status. *ERBB2* gene amplification was not included in the multivariate analyses since 42 of the primary tumours were lacking *ERBB2* data. *P*-values $\leqslant 0.05$ were considered statistically significant. All statistical analyses were performed using the BMDP statistical software package (Dixon *et al.*, 1990).

**Table I** *TP53* mutations and nuclear p53 protein accumulation

| *Pat. no.* | *CDGE/DGGE mutation in exon* | *Immunostaining* *PAb421* | *PAb1801* | *PAb240* | *NCL-CM1* |
|---|---|---|---|---|---|
| 3 | 8 | + | + + | + + | n.d. |
| 16 | 8 | + | + | + | n.d. |
| 20 | 5 | + | + + | – | n.d. |
| 22 | 5 | n.d. | n.d. | n.d. | n.d. |
| 24 | 7 | – | + | – | n.d. |
| 29 | 5<br>8 | – | + | – | n.d. |
| 34* | 5 | – | + | – | + |
| 50 | 7 | – | + | – | + + |
| 59 | 5 | + + | + + + | + + | n.d. |
| 65 | 8 | + | + + | – | n.d. |
| 71 | 7 | – | – | – | n.d. |
| 78* | 6 | n.d. | – | n.d. | – |
| 83 | 5 | + | + | – | n.d. |
| 88 | 8 | – | + | + + | n.d. |
| 101 | 5 | – | + | – | n.d. |
| 104 | 7 | + | + + | – | n.d. |
| 106 | 7 | + + + | + + + | + + + | n.d. |
| 111 | 6 | + | + + | + | n.d. |
| 112 | 7 | + + | – | + | n.d. |
| 115* | – | + | + | – | n.d. |
| 118 | 8 | n.d. | – | n.d. | – |
| 119 | 8 | + | + + + | + | n.d. |
| 120 | 6 | + + | + + + | + | n.d. |
| 132* | 7 | n.d. | + + + | n.d. | + + + |
| 135 | 7 | – | – | – | n.d. |
| 160 | 7 | n.d. | + | n.d. | + |
| 161 | 7 | n.d. | + + + | n.d. | + + + |
| 181 | 8 | n.d. | – | n.d. | + |
| 193* | 5 | n.d. | + | n.d. | + + |
| 208 | 5 | n.d. | + | n.d. | + + |
| 223 | – | n.d. | – | n.d. | + |
| 228 | 8 | n.d. | – | n.d. | – |
| 315 | – | n.d. | + | n.d. | + + |
| 318* | 8 | n.d. | + + | n.d. | + + + |
| 343 | – | n.d. | + + | n.d. | + + + |
| 353 | – | n.d. | + | n.d. | + |
| 359 | 7 | n.d. | – | n.d. | + |
| 369 | – | n.d. | + | n.d. | – |
| 374 | 8 | n.d. | + + | n.d. | + + + |
| 400 | 7 | n.d. | + + | n.d. | + + + |
| 406 | 5 | n.d. | + + + | n.d. | + + + |
| 416 | 5 | n.d. | + | n.d. | + |
| 418 | 5 | n.d. | – | n.d. | – |
| 434 | 8 | n.d. | – | n.d. | – |
| 440 | 5 | n.d. | + + | n.d. | + + |
| 443 | – | n.d. | + + + | n.d. | + + + |
| 449 | 8 | n.d. | – | n.d. | + + |

* = metastatic tissue. n.d. – not done, suitable material not available. – = no cells with immunoreactive nucleus. + = 0–5% cells with immunoreactive nucleus. + + = 5 R 50% cells with immunoreactive nucleus. + + + = 50% cells with immunoreactive nucleus.

**Table II** Location of and specific sequence alterations for the TP53 mutations

| *Pat. no.* | *Exon no.* | *codons no.* | *Sequence change* | *Aminoacid change* | *Nucleotide change* |
|---|---|---|---|---|---|
| 3 | 8 | 285 | GAG →AAG | Glu →Lys | G:C →A:T |
| 16 | 8 | 273 | CGT →CAT | Arg →His | G:C →A:T |
| 20 | 5 | 174-180 | AGG...GAG →AGAG | Frameshift | 17 bp deleted |
| 22 | 5 | 172-173 | GTT GTG →GTGTG | Frameshift | T:A bp deleted |
| 24 | 7 | 248 | CGG →CAG | Arg →Gln | G:C →A:T |
| 29 | 8 | 273 | CGT →CAT | Arg →His | G:C →A:T |
| 29 | 5 | 128 | CCT →TCT | Pro →Ser | C:G →T:A |
| 34* | 5 | 128 | CCT →CCG | Pro →Pro | T:A →G:C |
| 50 | 7 | 242 | TGC →TTC | Cys →Phe | G:C →T:A |
| 59 | 5 | 134 | TTT →CTT | Phe →Leu | T:A →C:G |
| 65 | 8 | 273 | CGT →CAT | Arg →His | G:C →A:T |
| 71 | 7 | 251-252 | ATC CTC →ATCTC | Frameshift | C:G bp deleted |
| 78* | 6 | 204 | GAG →TAG | Glu →Stop | G:C →T:A |
| 83 | 5 | 175 | CGC →CAC | Arg →His | G:C →A:T |
| 88 | 8 | 274 | GTT →GCT | Val →Ala | T:A →C:G |
| 101 | 5 | 181 | CGC →CAC | Arg →His | G:C →A:T |
| 104 | 7 | 239 | AAC →ACC | Asn →Thr | A:T →C:G |
| 106 | 7 | 238 | TGT →TTT | Cys →Phe | G:C →T:A |
| 111 | 6 | 195 | ATC →ACC | Ile →Thr | T:A →C:G |
| 112 | 7 | 248 | CGG →TGG | Arg →Trp | C:G →T:A |
| 118 | 8 | – | | | |
| 119 | 8 | 281 | GAC →GGC | Asp →Gly | A:T →G:C |
| 120 | 6 | 194 | CTT →CGT | Leu →Arg | T:A →G:C |
| 132* | 7 | 248 | CGG →CAG | Arg →Gln | G:C →A:T |
| 135 | 7 | 244-247 | GGC...AAC →GAAC | Frameshift | 8 bp deleted |
| 160 | 7 | 248 | CGG →TGG | Arg →Trp | C:G →T:A |
| 161 | 7 | 245 | GGC →AGC | Gly →Ser | G:C →A:T |
| 181 | 8 | 282 | CGG →CAG | Arg →Gln | G:C →A:T |
| 193* | 5 | 174-180 | AGG...GAG →AGAG | Frameshift | 17 bp deleted |
| 208 | 5 | 179 | CAT →TAT | His →Tyr | C:G →T:A |
| 228 | 8 | – | | | |
| 318* | 8 | 282 | CGG →TGG | Arg →Trp | C:G →T:A |
| 359 | 7 | 242 | TGC →TAC | Cys →Tyr | G:C →A:T |
| 374 | 8 | 281 | GAC →CAC | Asp →His | G:C →C:G |
| 400 | 7 | 248 | CGG →CAG | Arg →Gln | G:C →A:T |
| 406 | 5 | – | | | |
| 416 | 5 | – | | | |
| 418 | 5 | 133-134 | ATG TTT →ATTTT | Frameshift | G:C bp deleted |
| 434 | 8 | 271 | GAG →TAG | Glu →Stop | G:C →T:A |
| 440 | 5 | – | | | |
| 449 | 8 | – | | | |

* = metastatic tissue

**Figure 1** **a**, Constant denaturant gel electrophoresis (CDGE) of PCR amplified exon 7 fragments from tumour no. 132 (bronchial metastasis) (lane 1), a known codon 248 mutant (CGG→TGG) (lane 2), and wild-type tumours (lanes 3-5). The 12.5% polyacrylamide gel contained 45% denaturant (100% denaturant corresponds to 7 M urea and 40% formamide) and was run for 2 h at 56°C at 80 V constant. **b**, PCR amplified exon 7 fragment of tumour no. 132 analysed on a 12.5% polyacrylamide gel with a 10-60% gradient of denaturant. The PCR product was loaded into a long well on the top of the gel and run with the electrophoresis direction perpendicular to the denaturant gradient for 2 h at 56°C at 80 V constant. **c**, Sequencing analysis of PCR amplified exon 7 of tumour no. 132. A CGG→CAG substitution is seen in codon 248.

### Results

### TP53 *mutation analyses*

The CDGE analyses demonstrated that 35 of the 163 primary tumours (22%) and 5 of the 16 metastases (31%) contained mutations in exons 5 through 8. One primary tumour contained two independent mutations (tumour no. 29, Table I). A representative CDGE gel is shown in Figure 1a. Samples that were suspected to harbour mutations were reamplified and submitted to DGGE (Figure 1b). PCR fragments that had a mobility different from normal DNA in the CDGE and DGGE were considered to be mutant. However, exon 6 fragments that had aberrant bands were not considered to be mutant if they contained the polymorphic *Taq*I site in codon 213.

The approximate position and nature of each mutation could be predicted from these gels. For instance, fragments that migrated more slowly than the wild-type were considered to have undergone G:C→A:T transitions, since such mutations result in the destabilizing loss of a hydrogen bond. The exon in which each mutation resided, is given in Table I. Thirteen mutations were found in exon 5, three in exon 6, twelve in exon 7, and thirteen in exon 8. Accordingly, mutations were most frequently observed in exon 7 (0.235 mutations/base pair screened in exon 7 *vs* 0.117, 0.076, and 0.037 mutations/base pair screened in exons 8, 5 and 6, respectively, $P = 0.0014$).

Samples that were mutant according to the melting gel analyses (CDGE and DGGE) were submitted to direct sequencing (Figure 1c and Table II). The sequence analyses failed to confirm mutations in six samples that were mutant according to the melting gels. These results were confirmed by reamplification and resubmission to CDGE, DGGE, and sequence analysis. It should be noted that the mutant bands were faint in three of the six tumours (tumours no. 118, 228 and 416), but that the heteroduplexes convincingly indicated mutations. Furthermore, four of the six samples had nuclear accumulation of p53 protein (tumours no. 406, 416, 440, and 449, Tables I and II).

Table II shows that 29 point mutations and six deletions were found by sequencing. Five point mutations were found in codon 248, three in codon 273, and two in codons 128, 281, and 282. The majority of the point mutations appeared at G:C base pairs (22 of 29, 76%). G:C→A:T transitions accounted for 17, G:C→T:A transversions for 4, and G:C→C:G transversions for 1 mutation (59, 14, and 4% of the point mutations, respectively). Four of the point mutations at T:A base pairs were T:A→G:C transversions and three were T:A→C:G transitions. The deletions consisted of three single base pair losses (tumours no. 22, 71, and 418), one 8 base pair loss (tumour no. 135), and two identical 17 base pair losses (tumours no. 20 and 193, involving codons 174–180).

Neither of the two tumours with stop codon creating mutations (Table II, no. 78 at codon 204 and no. 434 at codon 271) contained cells with nuclear p53 protein accumulation, whereas the only tumour with a sense mutation (Table II, tumour no. 34, codon 128) surprisingly had p53 protein immunopositive cells.

### *p53 immunostaining*

Immunohistochemistry demonstrated that 35 of 162 primary carcinomas (22%) and four of 15 metastases (27%) contained cells with nuclear p53 protein accumulation (Table 1). The p53 protein immunopositive tumours exhibited diffuse or granular nuclear staining. Representative positive stainings are shown in Figure 2a–c. No immunoreactivity was seen in the normal breast tissue surrounding the tumours, but in a number of cases PAb 240 gave nonspecific binding in connective tissue. These samples were scored as negative.

Twenty-two tumours were analysed with PAb1801 on frozen as well as on paraffin embedded tissue. Ten of these samples were scored as positive and nine as negative on both frozen and paraffin embedded tissue, whereas three samples gave positive staining only on paraffin embedded tissue.

The association between *TP53* mutation and p53 protein accumulation was highly significant. Among the 177 tumours that were analysed for both kind of alterations, nuclear p53 protein accumulation was detected in 32 of 39 mutated and seven of 138 unmutated samples ($P < 0.00001$). In total 42 of 163 primary tumours (26%) and five of 16 metastases (31%) had some kind of *TP53* alteration.

### *Relationship to clinical and histopathological parameters*

Table III gives the relationships between *TP53* alterations in primary tumour and several clinical and histopathological parameters, including *ERBB2* gene amplification. *TP53* alterations were significantly more often found in ductal tumours, tumours with T-status larger than 1, node positive tumours, oestrogen receptor negative tumours, progesterone receptor negative tumours, tumours with *ERBB2* gene amplification, and invasive ductal carcinomas.

### *Univariate survival analyses*

Univariate analyses (Mantel Cox tests) demonstrated significantly shorter overall and disease-free survival for patients who had primary tumours with *TP53* alterations (Table IV). *TP53* mutation was a somewhat weaker, but

**Table III** *TP53* alterations in relation to clinical and histopathological parameters

| | *TP53 mutation* | | | *p53 protein accumulation* | | | *TP53 mutation and/or p53 protein accumulation* | | |
|---|---|---|---|---|---|---|---|---|---|
| | *Altered/total* | | *Signif. level* | *Altered/total* | | *Signif. level* | *Altered/total* | | *Signif. level* |
| Histol. type | | | | | | | | | |
| Ductal | 31/127 | (24%) | | 32/127 | (25%) | | 38/127 | (30%) | |
| Others | 4/36 | (11%) | NS | 3/35 | ( 9%) | $P = 0.060$ | 4/36 | (11%) | $P = 0.039$ |
| T-status | | | | | | | | | |
| T = 1 | 9/76 | (12%) | | 12/76 | (16%) | | 13/76 | (17%) | |
| T > 1 | 26/87 | (30%) | $P = 0.009$ | 23/86 | (27%) | NS | 29/87 | (33%) | $P = 0.0290$ |
| Node status | | | | | | | | | |
| negative | 17/100 | (17%) | | 16/99 | (16%) | | 19/100 | (19%) | |
| positive | 18/59 | (31%) | $P = 0.074$ | 19/59 | (32%) | $P = 0.032$ | 23/59 | (39%) | $P = 0.010$ |
| Oestr. rec. | | | | | | | | | |
| positive | 13/109 | (12%) | | 14/109 | (13%) | | 16/109 | (15%) | |
| negative | 22/54 | (41%) | $P = 0.0001$ | 21/53 | (40%) | $P = 0.0002$ | 26/54 | (48%) | $P = 0.00001$ |
| Prog. rec. | | | | | | | | | |
| positive | 12/89 | (13%) | | 13/89 | (15%) | | 14/89 | (16%) | |
| negative | 23/74 | (31%) | $P = 0.011$ | 22/73 | (30%) | $P = 0.028$ | 28/74 | (38%) | $P = 0.002$ |
| ERBB2 | | | | | | | | | |
| amplif. | 10/23 | (43%) | | 11/23 | (48%) | | 11/23 | (48%) | |
| nonamplif. | 19/98 | (19%) | $P = 0.030$ | 16/97 | (16%) | $P = 0.003$ | 21/98 | (21%) | $P = 0.020$ |

**Figure 2** Tumour no. 106. The majority of tumour cells show strong nuclear staining with the monoclonal p53 antibodies **a**, PAb 421, **b**, PAb 1801, and **c**, PAb 240 (× 580).

significant predictor of both overall and disease-free survival, and so was nuclear p53 protein accumulation. Node status represented the most powerful prognostic factor in these analyses. The node positive patients ($n = 59$) showed a trend towards reduced overall survival and had borderline significantly reduced disease-free survival if their primary tumours harboured *TP53* alterations, whereas the node negative patients ($n = 100$) showed non-significant trends towards reduced overall and disease-free survival if they had *TP53* alterations (Figure 3a and b).

### *Multivariate survival analyses*

The multivariate analyses selected node status and *TP53* alterations as significant variables for predicting overall survival, whereas node status, *TP53* alterations, and T-status

**Figure 3** **a**, Overall survival curves calculated with the life table method by node status and *TP53* alterations. A: node neg., *TP53* neg. B: node neg., *TP53* pos. C: node pos., *TP53* neg. D: node pos., *TP53* pos. Results of pairwise comparisons of the curves based on the Mantel cox test, A *vs* B, $P = 0.130$; C *vs* D, $P = 0.100$. **b**, Disease free survival curves calculated with the life table method by node status and *TP53* alterations. A: node neg., *TP53* neg. B: node neg., *TP53* pos. C: node pos., *TP53* neg. D: node pos., *TP53* pos. Results of pairwise comparisons of the curves based on the Mantel cox test, A *vs* B, $P = 0.170$; C *vs* D, $P = 0.050$.

were selected as significant variables for predicting disease-free survival (Table Va and b). When substituting *TP53* mutation or nuclear p53 protein accumulation for *TP53* alterations in the models, these factors were weaker, but still statistically significant independent prognostic parameters for predicting disease-free survival ($P = 0.023$ and $P = 0.036$, respectively). In the overall survival analysis *TP53* mutations were of borderline significance ($P = 0.051$), whereas p53 protein accumulation not turned out to be of independent prognostic relevance ($P = 0.200$).

### Discussion

The present work demonstrates *TP53* mutations in exons 5 through 8 in 21% of the primary breast carcinomas. The frequency has previously been reported to be 17–46% (Kovach *et al.*, 1991; Osborne *et al.*, 1991; Runnenbaum *et al.*, 1991; Coles *et al.*, 1992; Mazars *et al.*, 1992; Thorlacius *et*

**Table IV** Univariate analyses of overall and disease-free survival

| *Factor* | *Overall survival* | *Disease-free survival* |
|---|---|---|
| Node status | $P < 0.0001$ | $P < 0.0001$ |
| *TP53* alteration | $P = 0.0005$ | $P = 0.0002$ |
| *TP53* mutation | $P = 0.002$ | $P = 0.001$ |
| p53 overexpression | $P = 0.013$ | $P = 0.002$ |
| *ERBB2* gene amplification | $P = 0.008$ | $P = 0.0001$ |
| T-status | $P = 0.046$ | $P = 0.0003$ |
| Oestrogen rec. status | $P = 0.046$ | $P = 0.055$ |
| Progesterone rec. status | $P = 0.049$ | $P = 0.027$ |

**Table Va** Independent prognostic parameters selected by multivariate overall survival analysis

| *Variable* | *P-value* | *Relative hazard* |
|---|---|---|
| Node status | <0.0001 | 7.3592 |
| *TP53* alteration | 0.0163 | 2.9476 |

**Table Vb** Independent prognostic parameters selected by multivariate disease-free survival analysis

| *Variable* | *P-value* | *Relative hazard* |
|---|---|---|
| Node status | $<0.0001$ | 6.3561 |
| *TP53* alteration | 0.0099 | 2.3128 |
| T-status $>1$ | 0.0227 | 1.4516 |

*al.*, 1993). The varying frequencies might be due to differences in the proportion of early stage patients in the populations studied. Furthermore, it might reflect that different regions of the gene have been screened for mutations. In the present work mutations were observed more frequently in exon 7 (codons 237–253) than in exons 5, 6 and 8 (mutations/base pair screened). This observation is particularly interesting considering the fact that parts of exon 7 are extremely well conserved throughout the evolution. The 17 amino acid long stretch encoded by codons 237–253 has been demonstrated to be identical in rainbow trout, xenopus, chicken, rat, mouse, monkey, and human (Soussi *et al.*, 1990).

Six of the mutations detected by CDGE and DGGE were not confirmed by the sequence analysis. The fact that four of these tumours had nuclear accumulation of p53 protein makes it reasonable to believe that the discrepancy is caused by a lower sensitivity of the sequence analysis than that of the CDGE. It should be noted that the mutant bands were faint in three of the six samples, making the identification of the mutations relyant on the presence of heteroduplexes. The heteroduplexes that easily are recognised in melting gels enable detection of mutations when present in 5% or more of the cell population (Hovig *et al.*, 1992).

In the present work 76% of the point mutations (22/29) were found at G:C base pairs even though the overall G:C content in the *TP53* gene not is higher than 56% (Coles *et al.*, 1992). This excess is in accordance with what has been reported by others (Hollstein *et al.*, 1991; Coles *et al.*, 1992; de Fromentel & Soussi, 1992). The nucleoside guanosine has been reported to be a preferential target for most chemical carcinogens (Kriek *et al.*, 1984). CpG nucleotides are preferentially thought to be involved in spontaneous mutations (de Fromentel & Soussi, 1992). In the present work codons 175, 181, 248, 273, and 282 (CGN) that encode arginine accounted for 12 of the 22 mutations in G:C base pairs (55%).

The majority of the point mutations were G:C→A:T transitions (59%). This frequency is somewhat above the 40% reported in breast carcinomas by Hollstein *et al.* (1991), who further reported the frequency in colon carcinomas and brain tumours to be 79 and 75%, respectively. G:C→T:A transversions account for 14% of the point mutations in our breast carcinomas. It should be noted that such transversions never have been found in colon carcinomas, but that they occur at a high frequency in non small cell lung cancer (57%), liver cancer (74%), and oesophageal cancer (24%), which are associated with specific mutagenic factors (Hollstein *et al.*, 1991). The G:C→T:A transversions observed in sporadic breast carcinomas might indicate that external or internal produced carcinogens take part in the development of these tumours.

The identification of nuclear accumulation of p53 protein in 22% of the primary breast carcinomas in the present study is significantly less than the 57% reported by Thompson *et al.* (1990) at the mRNA level, the 54% reported by Bartek *et al.*, (1990*a*) using PAb 1801, 240, and 421, and the 45% reported by Cattoretti *et al.* (1988) using PAb 1801. On the other end of the scale positive p53 protein immunostainings have been reported in 22 and 24% of primary breast carcinomas (Crawford *et al.*, 1984; Davidoff *et al.*, 1991*b*; Thor *et al.*, 1992). The varying frequencies can be explained by differences in the proportion of early stage tumours, by varying quality of the antibodies used, by systematical differences in interpretation, and by the observation that frozen samples that have been stored over several years show a lower frequency of positive reactions (Bartek *et al.*, 1990*a*). In accordance with Cattoretti *et al.* (1988), but in contrast to Davidoff *et al.* (1991*a,b*) we interpreted cases with only few nuclei immunoreactive for p53 protein as immunostaining positive. Several tumours with positive nuclear staining in rare cells were demonstrated to contain mutations in the present study, supporting the interpretation that these tumours did contain accumulated p53 protein.

In agreement with Cattoretti *et al.* (1988) and Bartek *et al.* (1990*b*) we found a discrepancy between the immunoreactivity of the different p53 protein monoclonal antibodies. This could be explained by varying affinity of the antibodies and by differences in the availability of the epitopes when the different mutant p53 proteins complex with other proteins (Yewdell *et al.*, 1986).

The strong association between presence of *TP53* mutations and presence of nuclear p53 protein accumulation ($P<0.00001$) is in agreement with previous reports (Bartek *et al.*, 1990*b*; Davidoff *et al.*, 1991*b*), and fits the observation that mutant p53 protein has a half-life exceeding that of wild-type protein (Nigro *et al.*, 1989). It furthermore indicates that nuclear p53 protein accumulation in most cases is due to mutations within the gene.

Mutations were not detected in 7 of the 39 tumours that were p53 protein immunopositive. These tumours might contain mutations outside the screened regions of the gene, or they might contain insufficient amounts of mutant cells. Furthermore, some of the tumours might have alternative mechanisms of *TP53* inactivation, such as *MDM2* gene amplification (Momand *et al.*, 1992; Oliner *et al.*, 1992) or alterations in *TP53* regulatory sequences. Recently, two affected individuals in a cancer prone family were reported to have high levels of p53 protein constitutively expressed in their normal epithelial, endothelial, and stromal cells (Barnes *et al.*, 1992). *TP53* was not mutated in these patients, suggesting that mutation in another gene was causing both the high level of p53 protein and the increased risk of neoplasia in this family.

The fact that 7 of 39 samples with mutations were immunostaining negative can be explained by presence of sense- and stop codon mutations (shown for tumours no. 78 and 434, Tables I and II), by frameshift mutations that give lead to an abnormal aminoacid sequence or to stop codons down the line, and by the possibility that not all amino acid substitutions lead to stabile complex formation resulting in increased half-life. Furthermore, insufficient quality of the frozen tissue subjected to analysis might lead to false negative results.

The present work demonstrates that *TP53* alterations are associated with several clinical and histopathological parameters previously demonstrated to be of prognostic relevance (Table III). The observation that *TP53* alterations were detected more frequently in oestrogen and progesterone receptor negative tumours confirms previous findings (Thompson *et al.*, 1990; Iwaya *et al.*, 1991; Ostrowsky *et al.*, 1991; Varley *et al.*, 1991; Isola *et al.*, 1992; Thor *et al.*, 1992), and might reflect lack of differentiation of the breast carcinoma cells.

Recently, wild-type p53 has been postulated to be important in holding up the cell to repair DNA damage (Levine *et al.*, 1991; Kuerbitz *et al.*, 1992; Lane, 1992; Vogelstein & Kinzler, 1992). Tumour cells in which *TP53* is inactivated do not switch off replication in order to allow extra time for repair. This might explain the observation that primary tumours with *TP53* alterations frequently also contain *ERBB2* gene amplification (Table III) (Horak *et al.*, 1991; Mazars *et al.*, 1992).

Our data demonstrate that *TP53* alterations in primary breast carcinomas may represent an independent prognostic parameter for predicting disease-free and overall survival, but any conclusions based on the multivariate survival analyses should be drawn with caution. The prognostic relevance was most pronounced when the data on mutations and protein accumulation were combined. This probably reflects that neither mutational analysis nor immunohistochemistry alone identifies all *TP53* tumour suppressor inactivated tumours.

Node status is the most important established prognostic factor permitting identification of breast cancer patients at risk of relapse. Nevertheless, 20–30% of the node negative patients do relapse (McGuire, 1989). Use of tumour markers to predict unfavourable prognosis independent of node status could permit the identification of a subset of node negative breast carcinoma patients that would benefit from adjuvant treatment. During the last decade several molecular and cellular markers have been proposed as possible prognostic indicators (McGuire *et al.*, 1992). In the present material the node negative patients with *TP53* alterations showed trends towards shortened, although not statistically significant reduced survival (Figure 3a and b). Further studies will be required to fully evaluate a possible relevance of *TP53* alterations in node negative breast carcinoma.

Sigrid Lystad, Merete Hektoen, Ellen Hellesylt, and Mette Myre are gratefully acknowledged for excellent technical assistance. T.I.A. and K.R.H. are research fellows of the Norwegian Cancer Society. This study was supported by grants from the Norwegian Cancer Society and Grete Harbitz legacy.

### References

BARNES, D.M., HANBY, A.M., GILLETT, C.E., MOHAMMED, S., HODGSON, S., BOBROW, L.G., LEIGH, I.M., PURKIS, T., MACGEOCH, S., SPURR, N.K., BARTEK, J., VOJTESEK, B., PICKSLEY, S.M. & LANE, D.P. (1992). Abnormal expression of wild type p53 protein in normal cells of a cancer family patient. *Lancet*, **340**, 259–263.

BARTEK, J., BARTKOVA, J., VOJTESEK, B., STASKOVA, Z., REJTHAR, A., KOVARIK, J. & LANE, D.P. (1990*a*). Patterns of expression of the p53 tumour suppressor in human breast tissues and tumours *in situ* and *in vitro*. *Int. J. Cancer*, **46**, 839–844.

BARTEK, J., IGGO, R., GANNON, J. & LANE, D.P. (1990*b*). Genetic and immunochemical analysis of mutant p53 in human breast cancer cell lines. *Oncogene*, **5**, 893–899.

BEAHRS, O.H., HENSON, D.E., HUTTER, R.V.P. & MYERS, M.H. (eds). (1988). *Manual for Staging of Cancer*. 3rd. ed. p. 145. J.P. Lippincott: Philadelphia.

BØRRESEN, A.-L., OTTESTAD, L., GAUSTAD, A., ANDERSEN, T.I., HEIKKILÄ, R., JAHNSEN, T., TVEIT, K. & BØRRESEN, A.-L. (1990). Amplification and protein over-expression of the *neu*/HER-2/c-*erbB*-2 protooncogene in human breast carcinomas: relationship to loss of gene sequences on chromosome 17, family history and prognosis. *Br. J. Cancer*, **62**, 585–590.

BØRRESEN, A.-L., HOVIG, E., SMITH-SØRENSEN, B., MALKIN, D., LYSTAD, S., ANDERSEN, T.I., NESLAND, J.M., ISSELBACHER, K.J. & FRIEND, S.H. (1991). Constant denaturant gel electrophoresis as a rapid screening technique for p53 mutations. *Proc. Natl Acad. Sci. USA*, **88**, 8405–8409.

BØRRESEN, A.-L., ANDERSEN, T.I., GARBER, J., PIRAUX, N.B., THORLACIUS, S., EYFJÖRD, J., OTTESTAD, L., SMITH-SØRENSEN, B., HOVIG, E., MALKIN, D. & FRIEND, S.H. (1992). Screening for germ-line *TP53* mutations in breast cancer patients. *Cancer Res.*, **52**, 3234–3236.

CATTORETTI, G., RILKE, F., ANDREOLA, S., D'AMATO, L. & DELIA, D. (1988). P53 expression in breast cancer. *Int. J. Cancer*, **41**, 178–183.

COLES, C., CONDIE, A., CHETTY, U., STEEL, M., EVANS, H.J. & PROSSER, J. (1992). p53 mutations in breast cancer. *Cancer Res.*, **52**, 5291–5298.

CONDIE, A., EELES, R., BØRRESEN, A.-L., COLES, C., COOPER, C. & PROSSER, J. (1993). Detection of point mutations in the p53 gene: comparison of single-strand conformation polymorphism, constant denaturant gel electrophoresis, and hydroxylamine and osmium tetroxide techniques. *Human Mut.*, **2**, 58–66.

COX, D.R. (1972). Regression models and lifetables. *J. Royal Stat. Soc. B.*, **34**, 187–220.

CRAWFORD, L.V., PIM, D.C. & LAMB, P. (1984). The cellular protein p53 in human tumours. *Mol. Biol. Med.*, **2**, 261–272.

DAVIDOFF, A.M., HERNDON, J.E., GLOVER, N.S., KERNS, B.-J.M., PENCE, J.C., IGLEHART, J.D. & MARKS, J.R. (1991*a*). Relation between p53 overexpression and established prognostic factors in breast cancer. *Surgery*, **110**, 259–264.

DAVIDOFF, A.M., HUMPHREY, P.A., IGLEHART, J.D. & MARKS, J.R. (1991*b*). Genetic basis for p53 overexpression in human breast cancer. *Proc. Natl Acad. Sci. USA*, **88**, 5006–5010.

DEFROMENTEL, C.C. & SOUSSI, T. (1992). *TP53* tumor suppressor gene: a model for investigating human mutagenesis. *Genes, Chrom. Cancer*, **4**, 1–15.

DIXON, W.J., BROWN, M.B., ENGELMAN, L. & JENRICH, R.I. (eds). (1990). *BMDP Statistical Software Manual*. Univ. of Calif. Press.: Berkely.

HINDS, P.W., FINLAY, C.A., QUARTIN, R.S., BAKER, S.J., FEARON, E.R., VOGELSTEIN, B. & LEVINE, A.J. (1990). Mutant p53 DNA clones from human colon carcinomas cooperate with *ras* in transforming primary rat cells: a comparison of the 'hot spot' mutant phenotypes. *Cell Growth Different.*, **1**, 571–580.

HOLLSTEIN, M., SIDRANSKY, D., VOGELSTEIN, B. & HARRIS, C.C. (1991). p53 mutations in human cancers. *Science*, **253**, 49–53.

HORAK, E., SMITH, K., BROMLEY, L., LEJEUNE, S., GREENALL, M., LANE, D. & HARRIS, A.L. (1991). Mutant p53, EGF receptor and c-erbB-2 expression in human breast cancer. *Oncogene*, **6**, 2277–2284.

HOVIG, E., SMITH-SØRENSEN, B., GEBHARDT, M.C., RYBERG, D., LOTHE, R. & BØRRESEN, A.-L. (1992). No alterations in exon 21 of the *RB1* gene in sarcomas and carcinomas of the breast, colon and lung. *Genes, Chrom. Cancer*, **5**, 97–103.

HSU, S.-M., RAINE, L. & FANGER, H. (1981). A comparative study of the peroxidase-antiperoxidase method and an avidin-biotin complex method for studying polypeptide hormones with radioimmunoassay antibodies. *Am. J. Clin. Pathol.*, **75**, 734–738.

IGGO, R., GATTER, K., BARTEK, J., LANE, D. & HARRIS, A.L. (1990). Increased expression of mutant forms of p53 oncogene in primary lung cancer. *Lancet*, **335**, 675–679.

ISOLA, J., VISAKORPI, T., HOLLI, K. & KALLIONEMI, O.P. (1992). Association of overexpression of tumor suppressor protein p53 with rapid cell proliferation and poor prognosis in node-negative breast cancer patients. *J. Natl Cancer Inst.*, **84**, 1109–1114.

IWAYA, K., TSUDA, H., HIRAIDE, H., TAMAKI, K., TAMAKUMA, S., FUKUTOMI, T., MUKAI, K. & HIROHASHI, S. (1991). Nuclear p53 immunoreaction associated with poor prognosis of breast cancer. *Jpn. J. Cancer Res.*, **82**, 835–840.

KOVACH, J.S., MCGOVERN, R.M., CASSADY, J.D., SWANSON, S.E., WOLD, L.E., VOGELSTEIN, B. & SOMMER, S.E. (1991). Direct sequencing from touch preparations of human carcinomas: analysis of p53 mutations in human breast carcinomas. *J. Natl Cancer Inst.*, **83**, 1004–1009.

KRIEK, E., ENGELSE, L.D., SCHERER, E. & WESTRA, J.G. (1984). Formation of DNA modifications by chemical carcinogens. Identification, localization and quantification. *Biochim. Biophys. Acta*, **738**, 181–201.

KUERBITZ, S.J., PLUNKETT, B.S., WALSH, W.V. & KASTAN, M.B. (1992). Wild-type p53 is a cell cycle checkpoint determinant following irradiation. *Proc. Natl Acad. Sci. USA*, **89**, 7491–7495.

KUNKEL, L.M., SMITH, K.D., BOYER, S.H., BORGAONKAR, D.S., WACHTEL, S.S., MILLER, O.J., BREG, W.R., JONES, H.W. Jr & RARY, J.M. (1977). Analysis of human Y-chromosome-specific reiterated DNA in chromosome variants. *Proc. Natl Acad. Sci. USA*, **3**, 1245–1249.

LEVINE, A.J., MOMAND, J. & FINLAY, C.A. (1991). The p53 tumour suppressor gene. *Nature*, **351**, 453–456.

LANE, D.P. (1992). p53, guardian of genome. *Nature*, **358**, 15–16.

MALKIN, D., LI, F.P., STRONG, L.C., FRAUMENI, J.F. Jr, NELSON, C.E., KIM, D.H., KASSEL, J., GRYKA, M.A., BISCHOFF, F.Z., TAINKSY, M.A. & FRIEND, S.H. (1990). Germ line p53 mutations in a familial syndrome of breast cancer, sarcomas, and other neoplasma. *Science*, **250**, 1233–1238.

MALKIN, D., JOLLY, K., PIRAUX, N.B., LOOK, A.T., STRONG, L.C., GEBHARDT, M.C., ANDERSEN, T.I., BØRRESEN, A.-L., LI, F.P., ZALLEN, J., GARBER, J., KABISCH, H., GREENBERG, M.L., PRATT, C., YANG, E. & FRIEND, S.H. (1992). Germline mutations of the p53 tumor-suppressor gene in children and young adults with second malignant neoplasms. *N. Engl. J. Med.*, **326**, 1309–1315.

MAZARS, R., SPINARDI, L., BENCHEIKH, M., SIMONY-LAFONTAINE, J., JEANTEUR, P. & THEILLET, C. (1992). *p53* mutations occur in aggressive breast cancer. *Cancer Res.*, **52**, 3918–3923.

MCGUIRE, W.L. (1989). Adjuvant therapy of node-negative breast cancer. *N. Engl. J. Med.*, **320**, 525–527.

McGUIRE, W.L., TANDON, A.K., ALLRED, D.C., CHAMNESS, G.C., RAVDIN, P.M. & CLARK, G.M. (1992). Prognosis and treatment decisions in patients with breast cancer without axillary node involvement. *Cancer*, **70**, 1775–1781.

MOLL, U.M., RIOU, G. & LEVINE, A.J. (1992). Two distinct mechanisms alter p53 in breast cancer: mutation and nuclear exclusion. *Proc. Natl Acad. Sci. USA*, **89**, 7262–7266.

MOMAND, J., ZAMBETTI, G.P., OLSON, D.C., GEORGE, D. & LEVINE, A.J. (1992). The *mdm-2* oncogene product forms a complex with the p53 protein and inhibits p53-mediated transactivation. *Cell*, **69**, 1237–1245.

NIGRO, J.M., BAKER, S.J., PREISINGER, A.C., JESSUP, J.M., HOSTETTER, R., CLEARY, K., BIGNER, S.H., DAVIDSON, N., BAYLIN, S., DEVILEE, P., GLOVER, T., COLLINS, F.S., WESTON, A., MODALI, R., HARRIS, C.C. & VOGELSTEIN, B. (1989). Mutations in the *p53* gene occur in diverse human tumor types. *Nature*, **342**, 705–708.

OLINER, J.D., KINZLER, K.W., MELTZER, P.S., GEORGE, D.L. & VOGELSTEIN, B. (1992). Amplification of a gene encoding a p53-associated protein in human sarcomas. *Nature*, **358**, 80–86.

OSBORNE, R.J., MERLO, G.R., MITSUDOMI, T., VENESIO, T., LISCIA, D.S., CAPPA, A.P.M., CHIBA, I., TAKAHASHI, T., NAU, M.M., CALLAHAN, R. & MINNA, J.D. (1991). Mutations in the *p53* gene in primary human breast cancers. *Cancer Res.*, **51**, 6194–6198.

OSTROWSKY, J.L., SAWAN, A., HENRY, L., WRIGHT, J., HENNESSY, H.C., LENNARD, T.J.W., ANGUS, B. & HORNE, C.H.W. (1991). p53 expression in human breast cancer related to survival and prognostic factors: an immunohistochemical study. *J. Pathol.*, **164**, 75–81.

OTTESTAD, L., ANDERSEN, T.I., NESLAND, J.M., SKREDE, M., TVEIT, K.M., NUSTAD, K. & BØRRESEN, A.-L. (1993). c-*erb*B-2, *int*-2, and c-*myc* gene amplification in node-negative breast carcinomas, relationship to prognosis. *Acta Oncol.*, (in press).

PROSSER, J., PORTER, D., COLES, C., CONDIE, A., THOMPSON, A.M., CHETTY, U., STEEL, C.M. & EVANS, H.J. (1992). Constitutional p53 mutation in a non-Li-Fraumeni cancer family. *Br. J. Cancer*, **65**, 527–528.

RUNNENBAUM, I.B., NAGARAJAN, M., BOWMAN, M., SOTO, D. & SUKUMAR, S. (1991). Mutations in p53 as potential molecular markers for human breast cancer. *Proc. Natl Acad. Sci. USA*, **88:** 10657–10661.

SERRA, A., GAIDANO, G.L., REVELLO, D., GUERRASIO, A., BALLERINI, P., DALLA FAVERA ?? & SAGLIO, G. (1992). A new TaqI polymorphism in the p53 gene. *Nucleic Acids Res.*, **20**, 928.

SIDERANSKY, D., TOKINO, T., HELZISOUER, K., RAUSCH, G., ZEHNBAUER, B., SHELTON, B., PRESTIGLACOMO, L., VOGELSTEIN, B. & DAVIDSON, N. (1992). Inherited p53 gene mutations in breast cancer. *Cancer Res.*, **52**, 2984–2986.

SMITH-SØRENSEN, B., GEBHARDT, M.C., KLOEN, P., AGUILAR, F., FRIEND, S.H. & BØRRESEN, A.-L. (1993). Screening for TP53 mutations in osteosarcomas using the polymerase chain reaction (PCR) in combination with constant denaturant gel electrophoresis (CDGE). *Human Mut.*, (in press).

SOUSSI, T., DE FROMENTEL, C.C. & MAY, P. (1990). Structural aspects of the p53 protein in relation to gene evolution. *Oncogene*, **5**, 945–952.

SRIVASTAVA, S., ZOU, Z., PIROLLO, K., BLATTNER, W. & CHANG, E.H. (1990). Germ line transmission of a mutated *p53* gene in a cancer-prone family with Li-Fraumeni syndrome. *Nature*, **348**, 747–749.

THOMPSON, A.M., STEEL, C.M., CHETTY, U., HAWKINS, R.A., MILLER, W.R., CARTER, D.C., FORREST, A.P.M. & EVANS, H.J. (1990). p53 gene mRNA expression and chromosome 17p allele loss in breast cancer. *Br. J. Cancer*, **61**, 74–78.

THOR, A.D., MOORE II, D.H., EDGERTON, S.M., KAWASAKI, E.S., REIHSAUS, E., LYNCH, H.T., MARCUS, J.N., SCHWARTZ, L., CHEN, L.-C., MAYALL, B.H. & SMITH, H.S. (1992). Accumulation of p53 tumor suppressor gene protein: an independent marker of prognosis in breast cancers. *J. Natl Cancer Inst.*, **84**, 845–855.

THORLACIUS, S., BØRRESEN, A.-L. & EYFJÖRD, J. (1993). Somatic P53 mutations in human breast carcinomas in an Icelandic population, a prognostic factor. *Cancer Res.*, **53**, 1637–1641.

VARLEY, J.M., BRAMMAR, W.J., LANE, D.P., SWALLOW, J.E., DOLAN, C. & WALKER, R.A. (1991). Loss of chromosome 17p13 sequences and mutation of p53 in human breast carcinomas. *Oncogene*, **6**, 413–421.

VOGELSTEIN, B. & KINZLER, K.W. (1992). p53 function and dysfunction. *Cell*, **70**, 523–526.

YEWDELL, J.W., GANNON, J.V. & LANE, D.P. (1986). Monocloncal antibody analysis of p53 expression in normal and transformed cells. *J. Virol.*, **59**, 444–452.